# Persister cells: formation, resuscitation and combative therapies

**DOI:** 10.1007/s00203-021-02585-z

**Published:** 2021-11-05

**Authors:** Jack Wainwright, Glyn Hobbs, Ismini Nakouti

**Affiliations:** Centre for Natural Products Discovery (CNPD), School of Pharmacy and Biomolecular Sciences, Liverpool John Moores University, Byrom Street, Liverpool, L3 3AF UK

**Keywords:** Persister cells, Resuscitation, Chemotaxis, Antimicrobials

## Abstract

Persister cells, or superfits, have been strongly implicated in the recalcitrance and recurrence of chronic bacterial infection through the dormant (metabolically reduced) phenotype they display and the tolerance to antimicrobial agents this dormancy grants them. The complex biochemical events that lead to the formation of persister cells are not completely understood, though much research has linked the degradation of type II toxin/antitoxin systems and reduced cellular ATP levels to the rise in stress response molecules (where (p)ppGpp is of particular interest), which induce this dormant state. The equally complex mechanism of resuscitation is initiated by the cells’ ability to sense nutrient availability via chemotaxis systems. Levels of secondary messenger proteins (i.e., cAMP) within the cell are reduced to allow the resuscitation of ribosomes, by ribosomal resuscitation factor HflX, to reinstate protein synthesis and, therefore, growth to re-populate. Techniques of superfit eradication utilise one, or more, of three approaches (i) direct killing, (ii) re-sensitising persister cells to conventional antimicrobials, or (iii) prevention of persister formation though few laboratory findings have been translated to clinical practice. This work will outline current findings in the field with a critical approach, where possible.

## Introduction

The ability of pathogenic bacteria to survive therapeutic intervention is quickly becoming a major medical crisis of the modern world, where the emergence of multidrug resistance amongst Gram-positive and -negative bacteria have rendered conventional antimicrobials inept (Frieri et al. 2017). Several resistance mechanisms have been recognised within bacterial populations including immunosuppression of the host, evasion of immune response by the pathogen and ineffectiveness of antibiotic therapies (Woudt et al. 2018). Persister cells are a survival mechanism exhibited by bacteria, during the exponential growth phase, in response to a range of adverse environmental conditions (Fisher et al. 2017). These superfits show tolerance rather than resistance through phenotypic differentiation to the general bacterial population; as there are no genotypic variations, the replenished post-stress population will display equal susceptibility to stressors as the original population (Nakouti et al. 2017). The role of persister cells has, in recent years, gained acknowledgement with regards to the recalcitrance and recurrence of bacterial infections, where antimicrobial therapy has been implemented (Fisher et al. 2017). The focus of this paper is to review research literature published around the phenomenon of persister cells, potential models for their formation, models for persister resuscitation that allows infection to recur and combatant therapies with the aim to increase efficacy of treatments targeted towards bacterial infections.

## From discovery to present day

For many years, the scope of research has been largely directed towards the resistance mechanisms of bacteria to antibiotics rather than the innate ability for subpopulation tolerance. While working with the still novel penicillin in 1944, Joseph Bigger, a medical doctor working in York, concluded that penicillin did not have the ability to completely sterilise infections (Bigger 1944). Bigger repeatedly observed growth following exposure of penicillin to *Staphylococcus pyogenes* and determined that the surviving cells must be in a “dormant, non-dividing state” being the first to refer to them as “persisters” (Bigger 1944). Up until the early 1990s, research focused heavily on bacterial resistance, until one Harris Moyed addressed persistence due to his interest in the non-heritable survival mechanisms of bacteria, which led to the discovery of potential models of persister formation (Lewis 2010).

## Persister cell dormancy

Persister cells are able to enter a dormant state with drastically reduced metabolic activity allowing them to survive exposure to antimicrobial drugs. Antibiotics rely on a living target to exert their bactericidal properties as they perform by corrupting the synthesis products of the bacteria, meaning the metabolism of the bacteria is required for effective antibiotic function (Kapoor et al. 2017). Dormancy of persisters, in the form of reduced protein synthesis, was evidenced by research conducted by Shah et al (2006), where a strain of *Escherichia coli* (*E. coli*)*,* which expressed Green Fluorescent Protein (GFP), was exposed to a β-lactam antibiotic (Shah et al. 2006). The strain used expressed GFP under control of a ribosomal promoter found only in dividing cells; after exposure to the antibiotic some cells appeared very faint fluorescence and were tolerant to ofloxacin, proving their status as persisters. However, limitations of the method were apparent; sorting the cells necessitates dilution of the culture into a buffer which changes the concentration and medium, therefore, promoting resuscitation of the original population from surviving persisters and decreasing persister levels (Lewis 2010).

Despite these limitations, when transcriptome analysis was performed it revealed a downregulation in biosynthesis genes and an increase in expression of several toxin/ antitoxin (TA) genes. The comparison between active gene profiles of persister cells and exponentially growing cells showed a clear decrease in the synthesis of proteins consistent with energy production and non-essential functions (e.g., chemotaxis), indicative of a state of dormancy (Shah et al. 2006). Although the dormancy state of persister cells has been demonstrated, there is little understanding on the mechanisms that occur to activate or promote this dormant state of persisters; nor which genes must remain active to eventually allow resuscitation of the bacterial population (Yamasaki et al. 2020).

## Models of persister cell formation

Research into possible models by which persister cell formation were induced, and the resulting tolerance towards bactericidal antibiotics, has been increasingly discussed in biofilm research over the last decade. The most popular models both determine that superfit formation occurs not solely as a result of direct exposure to antibiotics but rather as a response to common nutrient deficiencies experienced by pathogenic bacteria residing inside biofilms; with the added effect of conceiving tolerance within persister populations and the biofilms that contain them (Defraine et al. 2018).

### Type II toxin/antitoxin (TA) systems

TA systems refer to intricately linked genes of two (bicistronic) or more that are responsible for the encoding of a toxic protein and its corresponding antitoxin, either a secondary protein or RNA, which together form a neutral complex (Jankevicius et al. 2016). While the toxic protein is stable, the counteracting antitoxin is easily degradable by ATP dependent proteases; the toxins are often translation inhibitors, down regulating the synthesis of proteins through a variety of molecular mechanisms, hence their believed involvement in pathogenic bacterial dormancy and tolerance of antibiotics (Goormaghtigh et al. 2018).

The first type II TA system identified in vitro and to gain notoriety in the formation of persisters in the Gram-negative *E. coli* is the mutant high persistence gene A (*hipA*) and its upstream counterpart *hipB*, which together form the complex hipBA (Moyed and Bertrand 1983). In the scenario, where stress results in *hipA* levels greater than those of *hipB*, *hipA* is able to phosphorylate glutamate-tRNA (transfer RNA)–ligase (GltX) preventing the transfer of glutamate to tRNA^Glu^. The accumulation of uncharged tRNA^Glu^ in the ribosomal A site activates the ribosome-associated guanosine tetra- and pentaphosphate ((p)ppGpp) synthase, RelA; the resulting (p)ppGpp is believed to act as an alarmone and trigger the release of toxins from other TA systems (Semanjski et al. 2018). These toxins are then able to target other protein-encoding genes to downregulate synthesis to reduce metabolic activity and enter a state of dormancy. The same mutant *hipA* genes identified *in vitro* by Moyed in 1983 were found in patients with recurrent urinary tract infections (UTIs). In isolates of *Pseudomonas aeruginosa* from cystic fibrosis patients, and from patients with chronic *Candida albicans* infections further cementing the link between *hipA* and pathogenic recalcitrance (Conlon et al. 2016; LaFleur et al. 2006; Mulcahy et al. 2010; Schumacher et al. 2015).

In *E. coli*, ten other such TA systems have been identified, the toxins of which all behave as mRNA endonucleases (mRNases) and can be separated into superfamilies based on the cleaving point target of the mRNases (Maisonneuve et al. 2011). Six of these mRNases (RelE, YoeB, HigB, YhavV, YafO and YafQ) cleave mRNA at the ribosomal A site, whereas the other 4 (MazF, ChpB, MqsR and HicA) cleave RNA site—specifically and independently of the ribosome but all have the effect of downregulating protein translation (Maisonneuve et al. 2011). Evidence has shown that autoregulation of these TA bicistronic operons is controlled by the antitoxin, which binds to operator sequences in the TA promoter region; binding occurs more strongly when the antitoxin is in complex with the mRNase meaning they themselves behave as co-repressors of their own synthesis. However, mRNase in excess of its antitoxin disrupts TA complexes and promotes the upregulation of TA operon transcription (Overgaard et al. 2008). A proposed explanation for this state is activation of a stringent response in exposure to stress, namely, nutrient starvation, where a (p)ppGpp pathway involving a Long Form Filament (LON)-dependent mechanism leads to the degradation of antitoxins (Christensen-Dalsgaard et al. 2010).

Furthermore, (p)ppGpp of the stringent response is linked to the reduction of protein synthesis due it’s interaction with RNA polymerase. It is able to directly modulate transcription via activation of two key stress responses, the stress response for stationary phase (RpoS) and the stress response for misfolded proteins (RpoE), which leads to the reduction in ribosome synthesis (Song and Wood 2020). In addition to transcriptional modulation, (p)ppGpp stimulates the genes responsible for the production of Ribosome Modulation Factor (RMF), hibernation promoting factor (Hpf) and ribosome associated inhibitor (RaiA). RMF converts active 70S ribosomes into inactive 100S ribosomes via an inactive 90S dimer complex, Hpf converts 90S ribosomes into 100S ribosomes and RaiA inactivates 70S ribosomes leading to an inability for translation to occur (Song and Wood 2020).

A study performed by Maisonneuve (2011) showed that successive deletion of the ten type II TA systems in *E.coli* (known as the Δ10 strain) showed no exponential growth deficits, when compared to wild-strain *E.coli* but resulted in the reduction of persister levels following exposure to an antibiotic, evidencing their involvement in persistence (Maisonneuve et al. 2011). The study also concluded that the availability of LON is crucial to the ability of bacterial dormancy, as cells presenting LON deletion showed a drastic decrease in superfit levels when compared to strains mutated to display other protease deficiencies (Maisonneuve et al. 2011). This explanatory model influenced research for some time until the authors of the model discovered considerable φ80 phage infection of the original experiment, a “notorious laboratory contaminant” which could explain the decrease in persister levels and negate their conclusions of TA and LON involvement in the formation of persisters (Goormaghtigh et al. 2018). In a statement released by the authors of the study, it was explained that the claim of mRNase overproduction contributing to bacterial tolerance is still valid but that the data collected by the original study could no longer be used to support this model. Repeats of the methods in an environment free of φ80 phages, showed similar but far less conclusive results leaving the mechanism of persister formation in Gram-negative species in need of a great deal more research (Maisonneuve et al. 2011).

While the link between TA modules and persistence of Gram-negative bacteria has been studied, less is known about the mechanism of persistence in relation to Gram-positive populations. Similar deletion of type II TA systems in a sample of the Gram-positive *S. aureus*, as performed on the Gram-negative *E. coli*, showed no effect on the ability of superfits to arise (Conlon et al. 2016).

### Cellular ATP levels

Bactericidal antibiotics target metabolic pathways to target active sites; β-Lactams target peptidoglycan synthesis resulting in lysis, aminoglycosides target bacterial ribosomes and disrupt translation, and fluoroquinolones prevent re-ligation by DNA gyrase and topoisomerase leading to breakages in the double strand structure of bacterial DNA (Garneau-Tsodikova and Labby 2016; Idowu and Schweizer 2017; Kapoor et al. 2017). The ability of these pathways to occur is crucial for the exponential growth of the population and are dependent on ATP; suggesting that a reduction of available ATP may induce persister formation.

**Fig. 1:**
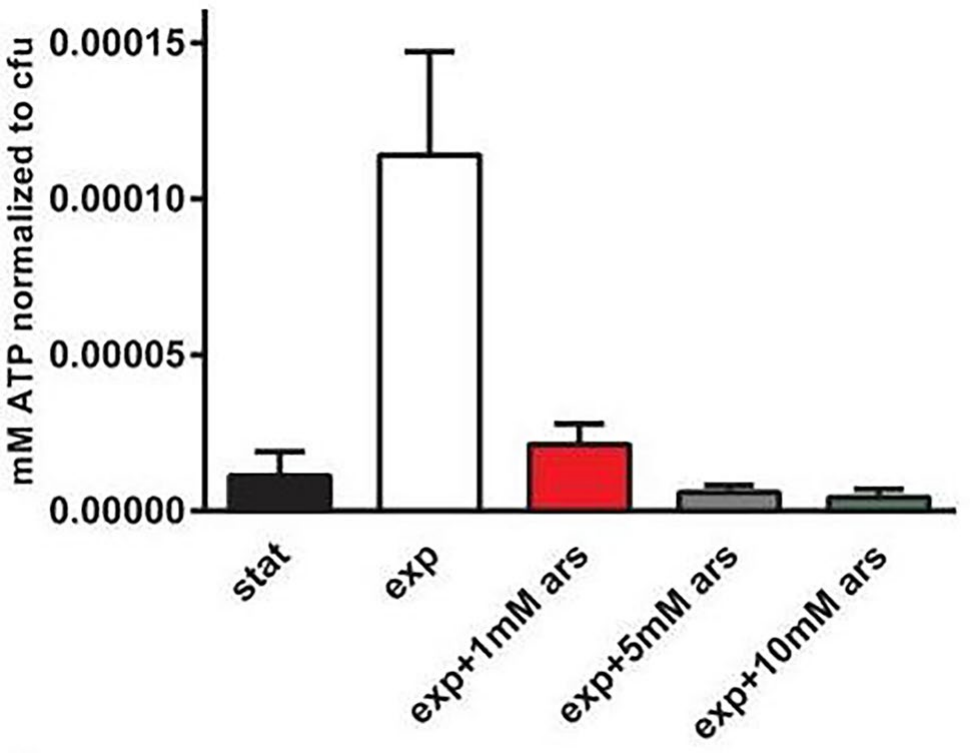
ATP levels of *S. aureus* in stationary and exponential growth phases; and in exponential phase following exposure to varying concentrations of Arsenate for 15 min. Arsenate forms rapidly hydrolysable ADP-As which reduce ATP availability. *Stat* stationary phase, *Exp* exponential phase, *Ars* arsenate. (Conlon et al. 2016)

A study by Conlon et al (2013) showed that ATP levels are significantly reduced in the stationary phase of *S. aureus* (Fig. 1); as the metabolic output of exponential growth requires larger concentrations of ATP (Conlon et al. 2013). Stationary phase is where prominent antibiotic tolerance similar to that of persisters is observed; suggesting that persister formation in *S. aureus* may be a result of the non-uniform entering of stationary phase (Conlon et al. 2016). Continuing this research in 2016, Conlon et al. found that treating growth phase *S. aureus* with arsenate, which substitutes the phosphate anion during glycolysis inhibiting ATP synthesis, to emulate the ATP levels of the stationary phase (Fig. 1) increased superfit formation by 325-fold compared to wild-type strains and that exposing samples to increased ATP levels decreased superfit formation 100-fold (Conlon et al. 2016; Kulshrestha et al. 2014). Several stationary phase specific promoters were found to have been activated in persisters of the reduced ATP samples (Conlon et al. 2016). Activation of these promoters suggests that it is the metabolic differences between the two growth phases imparting the ability for antibiotic evasion (Conlon et al. 2016).

Similar results were reported when the effect of cellular ATP levels were investigated in Gram-negative *E. coli* samples. Using the known persister reporter *rrnB* P1; which sense both (p)ppGpp and ATP; Shan et al (2017) discovered the continued reporting of persisters in both the Δ10TA strain and environments lacking (p)ppGpp (Shan et al. 2017). With findings consistent to those of the *S. aureus* experiment conducted by Conlon et al (2016), it could be proposed that while TA systems are apparent in persister formation, ATP levels play a universal role in the induction of persister cells across bacterial species (Conlon et al. 2016; Shan et al. 2017).

## Persister cell resuscitation

**Fig. 2:**
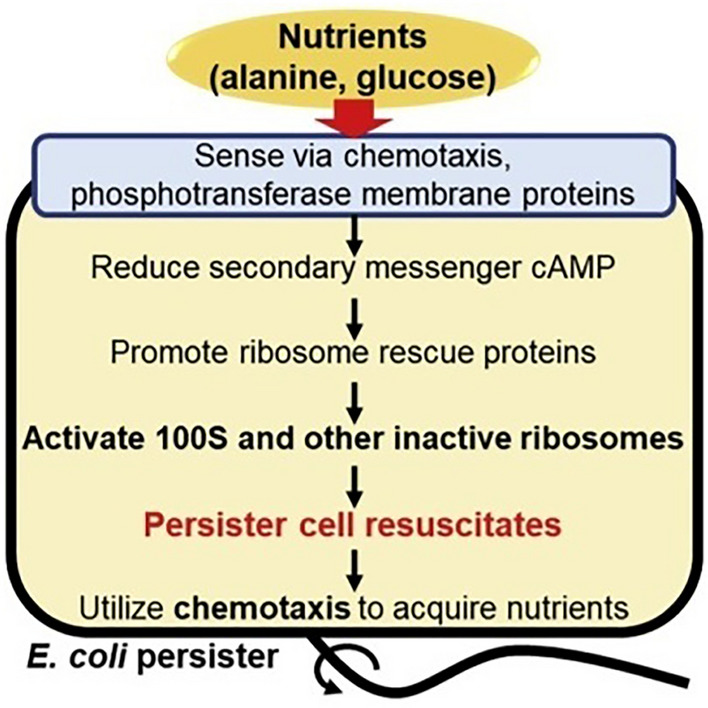
Suggested model for persister cell resuscitation, whereby the availability of nutrients in a post-stress environment promotes the return to normal metabolic function. Alanine and glucose are used as nutrient saurces (Yamasaki et al. 2020)

Regardless of the mechanisms which allow cells to enter the dormant state and become persisters, one thing is clear, that these superfits eventually return to a functional state, where the bacterial population is replenished and a relapse of infection can occur; i.e., the mechanisms that induce dormancy are reverted (Svenningsen et al. 2019). The exact process by which persister resuscitation occurs is not well known, though much research into the topic appears to have been conducted by Dr Ryota Yamasaki, and his team, in the recent years. His findings around persister cell waking in *E. coli* suggest that the complex process relies on the cell’s ability to sense nutrients (Fig. 2) rather than the previously hypothesised stochastic transition between phenotypes (Yamasaki et al. 2020).

### Nutrient signalling

The proven ability for persister cells to revive in response to advantageous environmental changes somewhat negates that revival from persister state is a random occurrence (Balaban et al. 2004; Kim et al. 2018). A study by Yamasaki et al (2020) hypothesised that the presence of single amino acids could trigger resuscitation (defined as growth or elongation within the sample), initial testing separated four groups of five amino acids, *E. coli* superfits were plated on agar plates in which these amino acids were the only carbon source and growth was observed after 6 h (Yamasaki et al. 2020). The reasoning behind this qualitative assessment method being that revived persisters grow at a similar rate to that of exponentially growing cells (Kim et al. 2018). Following this, individual amino acids were tested in the same manner and single cells were assessed using light microscopy; this showed alanine (Ala) as the amino acid that promoted the highest levels of waking (18 ± 1%, compared to asparagine (Asn) with 2 ± 2%); a negative control (agar lacking amino acids) showed no waking, proving the role of amino acids in persister cell resuscitation (Yamasaki et al. 2020).

Further to this, the study wanted to prove whether Ala acts as a true resuscitation signal, rather than simply being a carbon and energy source for growth, using an *E.coli* strain that contains an isogenic *dadA* mutation for inactivation of D-amino acid dehydrogenase to prevent growth in the presence of Ala; the study found that 4% of *dadA* superfits woke in the presence of Ala, whereas no waking was observed in the absence of Ala (Franklin and Venables 1976; Yamasaki et al. 2020). In addition, Ala was proven to wake superfits of the *dadA* strain even in the presence of a secondary carbon and energy source, pyruvate; pyruvate alone did not wake any single cells, whereas Ala was able to wake cells to the same extent when pyruvate was absent or present meaning it must act as a true signal in the resuscitation of persisters (Yamasaki et al. 2020).

### Chemotaxis systems

Motile bacteria use chemotaxis systems to migrate to environments more favourable to growth and survival, the genes for which are down regulated in the processes that result in cell dormancy (Bi and Sourjik 2018; Shah et al. 2006). By assessing how individual proteins affected the rate of waking within the persister population, Yamasaki et al (2020) found significant evidence for the role of chemotaxis proteins in persister resuscitation (Yamasaki et al. 2020). Using a collection of pCA24N-derived plasmids, for which each synthesises one *E.coli* protein (ASKA collection), persisters were formed and plated on Ala-containing plates with chloramphenicol to ensure plasmid retention (Yamasaki et al. 2020). After eight days, the largest colonies (i.e., the fastest growing) were purified and sequenced to reveal six proteins presumed to be stimulators of persister waking; single cells containing the ASKA plasmids for these proteins were observed on Ala agar gel pads; all six resulted in a rate of waking faster than the control containing an empty plasmid after an 18 h incubation period, with the greatest waking occurring in cells producing the chemotaxis response regulators CheY and CheA (with 100% and 82% of cells waking, respectively)(Yamasaki et al. 2020). In an effort to find more evidence, single cells of deletion mutant strains ΔCheY and ΔCheA were assessed and showed a complete inhibition in their waking; from here, all five of the methyl-accepting chemotaxis proteins (Tsr, Tar, Trg, Tap, and Aer) were investigated using isogenic mutants with Tar and Trg showing higher frequencies of waking when in conjunction with CheY and CheA (Yamasaki et al. 2020). Hence, the conclusion is drawn that persister resuscitation relies on Alanine signalling via the chemotaxis system, more specifically via the methyl-accepting chemotaxis proteins Tar and Trg, and the chemotaxis regulators CheY and CheA (Yamasaki et al. 2020).

### Ribosomal resuscitation via rescue proteins

In the formation of persisters, small positively signalling molecules regulate factors that, in high concentrations, result in the deactivation and hibernation of ribosomes and hence, the reduction of product synthesis consistent with dormancy (Gohara and Yap 2018). These signal molecules, cAMP and supposedly (p)ppGpp, are hypothesised to allow ribosomes revival when levels are decreased so that protein synthesis may resume (Song and Wood 2020; Yamasaki et al. 2020).

In short, elevated levels of cAMP have been linked to increased persistence due their participation in ribosomal protein gene regulation, including that of RMF (Shimada et al. 2013). Using single cells selected to produce elevated levels of cAMP, a clear reduction in rate of waking was observed (Yamasaki et al. 2020). Cells were also selected to produce the cAMP inhibitor CpdA, the addition of this inhibitor saw the rate of waking increase as did exposure to the cAMP inhibiting drug atropine with the greatest increase observed when both CpdA and atropine were used in synergy to decrease cAMP levels (Yamasaki et al. 2020). When comparing the ribosome resuscitation factor HflX to the ribosome rescue factor ArfB (a negative control as it is positively regulated by cAMP), cells producing HflX were able to wake much faster than control cells containing an empty plasmid and cells producing ArfB showed no waking; further, inactivating HflX resulted in the total inhibition of waking in single cells, while inactivating ArfB had no effect (Yamasaki et al. 2020).

With regards to (p)ppGpp, no discernible mechanism has been found to link it to the expression of HflX; however, it is known to bind to and inhibit the 100S splitting activity of HflX so that ribosomes remain in an inactive state (Yamasaki et al. 2020). When assessing the waking ability of mutated *E. coli* cells omitting RelA and spoT (a second (p)ppGpp synthase) no significant effects towards waking were noted; similar finding were reported when the same testing was performed on strain of *P. aeruginosa*, meaning (p)ppGpp does not play as large a role in cell resuscitation as previously hypothesised (Akiyama et al. 2017; Song and Wood 2020).

## Combatant therapies

**Fig. 3:**
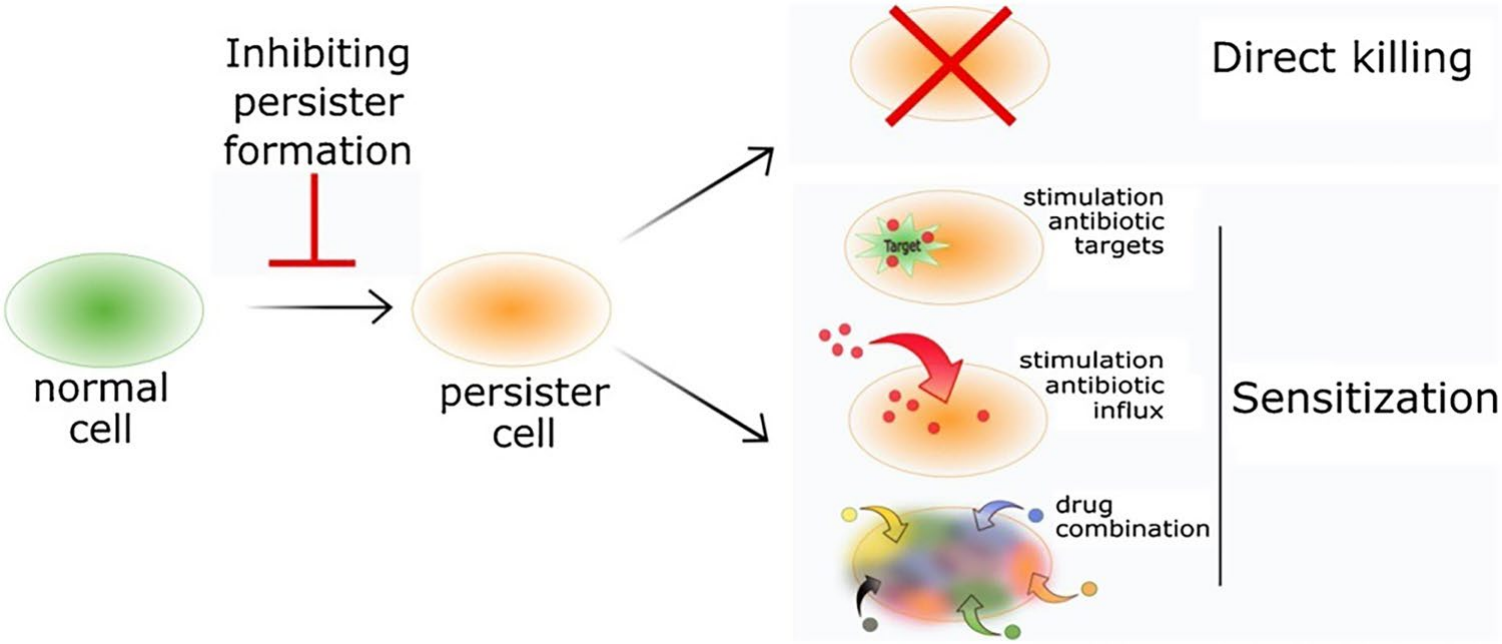
Schematic model for combatant persister cell therapies; one branch of treatment targets cells prior to persister formation, while the remaining two branches target existing persisters through either direct killing or sensitisation to conventional antibiotics by a number of mechanisms (Defraine et al. 2018)

Research into therapies capable of eradicating a persister population has provided an extensive list of molecules that with further research and development could be approved for clinical use; these molecules can be divided into three mechanisms of actions, where some molecules may overlap these categories, (i) direct killing of metabolically dormant persister cells, (ii) sensitising persisters to conventional antibiotics by promoting resuscitation and, (iii) compromising of molecules linked to the induction of persister cells (Fig. 3) (Defraine et al. 2018). It is important to recognise that in vivo, pathogenic bacteria often reside within biofilms so a difficulty in translating in vitro findings to effective clinical therapies is apparent (Wood, 2017).

### Direct killing

To directly target persister cells, interventions must utilise alternative targets to those of conventional antibiotics; suggested targets include, destruction of bacterial cell membrane, DNA cross-linking, inhibition of key enzymes and generation of reactive oxygen species (ROS) (Defraine et al. 2018). Targeting of bacterial cell walls is a logical approach as, despite dormancy, cells require an intact membrane to remain viable; both lipoglycopeptides and diarylquinolines, among other molecules, have shown promise in their ability to disrupt the bacterial membranes of persisters across multiple species of bacteria (Hurdle et al. 2011). Several existing anti-cancer drugs (e.g., Mitomycin C, Cisplatin and Anthracyclines) have been implicated in their ability to cross link DNA, even in notoriously difficult to treat species, though their high toxicity contraindicates their use in treatment (Cruz-Muñiz et al. 2017; Defraine et al. 2018). Periodic exposure to the antimicrobials might be required as persister cells are often located in the middle of a robust biofilm, making access to this particular sub-population very difficult. The exact therapy is dependent on bacterial kinetics.

### Sensitising persister cells

As antibiotic targets involve the corruption of synthesis products; one proposed method of exterminating persisters is to encourage resuscitation within the dormant population so that these targets are actively produced (Defraine et al. 2018). A number of molecules have been identified with the ability to stimulate the metabolism of persisters and cause a phenotypic switch from the tolerant dormant type to an antibiotic sensitive type (Kim et al. 2011; Pascoe et al. 2014). For antibiotics requiring active transport systems to exert their bactericidal properties, it has been proven possible to manipulate drug influx rates by generating a proton-motive force, modifying the membrane pH levels or by inhibiting amino acid synthesis to raise energy levels and increase uptake (Defraine et al. 2018; Duan et al. 2016). A final possible approach to sensitising persisters is to use a number of mechanistically different antibiotics; research has shown that subpopulations exist within a superfit faction that may exhibit sensitivity to different antibiotics, the implementation of existing clinical treatments may work to reduce treatment times as well as costs (Defraine et al. 2018). However, exposure to multiple antibiotics could risk promoting drug resistance.

### Preventing persister formation

The final strategy in combating persister cells lies in preventing persister formation in the first instance, by interfering with mechanisms known to occur in persister formation (Defraine et al. 2018). Targeting of the stringent response alarmone (p)ppGpp to prevent its accumulation has been shown to reduce persister formation across several Gram-positive species, as has inhibition of the SOS- and oxidative-stress responses (Defraine et al. 2018). Treatment of stationary phase cells with nitric oxide (NO) to inhibit respiration but prevent protein and RNA degradation produced populations that resumed growth when exposed to more favourable environments without the cells entering a state of dormancy; bypassing persister formation resulted in cells that remained susceptible to antibiotics, a finding that could be critical to preventing chronic infection (Orman and Brynildsen 2016). This approach will be particularly effective in wound healing.

## Conclusion

The tolerant behaviours of persister cells are arguably one of the most important phenotypes bacteria can possess for their survival, though their existence causes great difficulty in the treatment of pathogenic infections. Extensive research into both the formation and resuscitation of persister cells has provided insight into potential points of exploitation so as to improve the efficacy of treatment. The lack of consensus surrounding exact models of formation leaves open the opportunity for further research, both for *E. coli*, which has been the mainstay species for the majority of research conducted, and other species of bacteria prevalent in infections reported by clinicians. A much clearer understanding of the occurrences for which resuscitation necessitates is apparent when considering the work of Yamasaki et al (2020), whose detailed finding have answered many questions posed by the existence of persister cells (Yamasaki et al. 2020). A correlation between persisters and the emergence of resistant mutations within pathogenic species has also been observed, proving them to be a greater threat to modern healthcare efforts than may have previously been imagined and making any research into actionable anti-persister therapies that much more crucial (Windels et al. 2019).
